# Podoplanin: A potential therapeutic target for thrombotic diseases

**DOI:** 10.3389/fneur.2023.1118843

**Published:** 2023-03-09

**Authors:** Yaqian Huang, Manli Lu, Yi Wang, Chunyuan Zhang, Yongjun Cao, Xia Zhang

**Affiliations:** ^1^Department of Neurology, Clinical Research Center of Neurological Disease, The Second Affiliated Hospital of Soochow University, Suzhou, China; ^2^Department of Rehabilitation, The Second Affiliated Hospital of Soochow University, Suzhou, China

**Keywords:** podoplanin, thrombotic, inflammation, CLEC-2, platelet activation, epithelial-mesenchymal transition

## Abstract

As a specific lymphatic marker and a key ligand of C-type lectin-like receptor 2 (CLEC-2), podoplanin (Pdpn) is involved in various physiological and pathological processes such as growth and development, respiration, blood coagulation, lymphangiogenesis, angiogenesis, and inflammation. Thrombotic diseases constitute a major cause of disability and mortality in adults, in which thrombosis and inflammation play a crucial role. Recently, increasing evidence demonstrates the distribution and function of this glycoprotein in thrombotic diseases such as atherosclerosis, ischemic stroke, venous thrombosis, ischemic-reperfusion injury (IRI) of kidney and liver, and myocardial infarction. Evidence showed that after ischemia, Pdpn can be acquired over time by a heterogeneous cell population, which may not express Pdpn in normal conditions. In this review, the research progresses in understanding the roles and mechanisms of podoplanin in thromobotic diseases are summarized. The challenges of podoplanin-targeted approaches for disease prognosis and preventions are also discussed.

## Introduction

Podoplanin (Pdpn), named according to its expression in renal podocytes, is a type I transmembrane glycoprotein containing a large number of O-glycoside chains, which makes it a member of mucin-type proteins. Due to its expression in human and several mammal species in various cells and tissues, it has many different names. In human it is also called gp36 and T1α (1), however, in mice which is also known as Aggrus, OTS-8, gp38, and antigen PA2.26 (2–4). Pdpn is mainly involved in growth and development, respiration, blood coagulation, lymphangiogenesis, angiogenesis, and inflammation (5–7). Especially the interaction with its receptor C-type lectin-like receptor 2 (CLEC-2) has been shown to play an important role in thromboinflammation (8, 9). Pdpn expression is upregulated in both epithelial and mesenchymal cell compartments during thrombosis and inflammation, and a growing body of evidence indicates its prominence in these pathologies of thrombotic diseases.

## Structure, protein partners and cell expression

**Figure 1:**
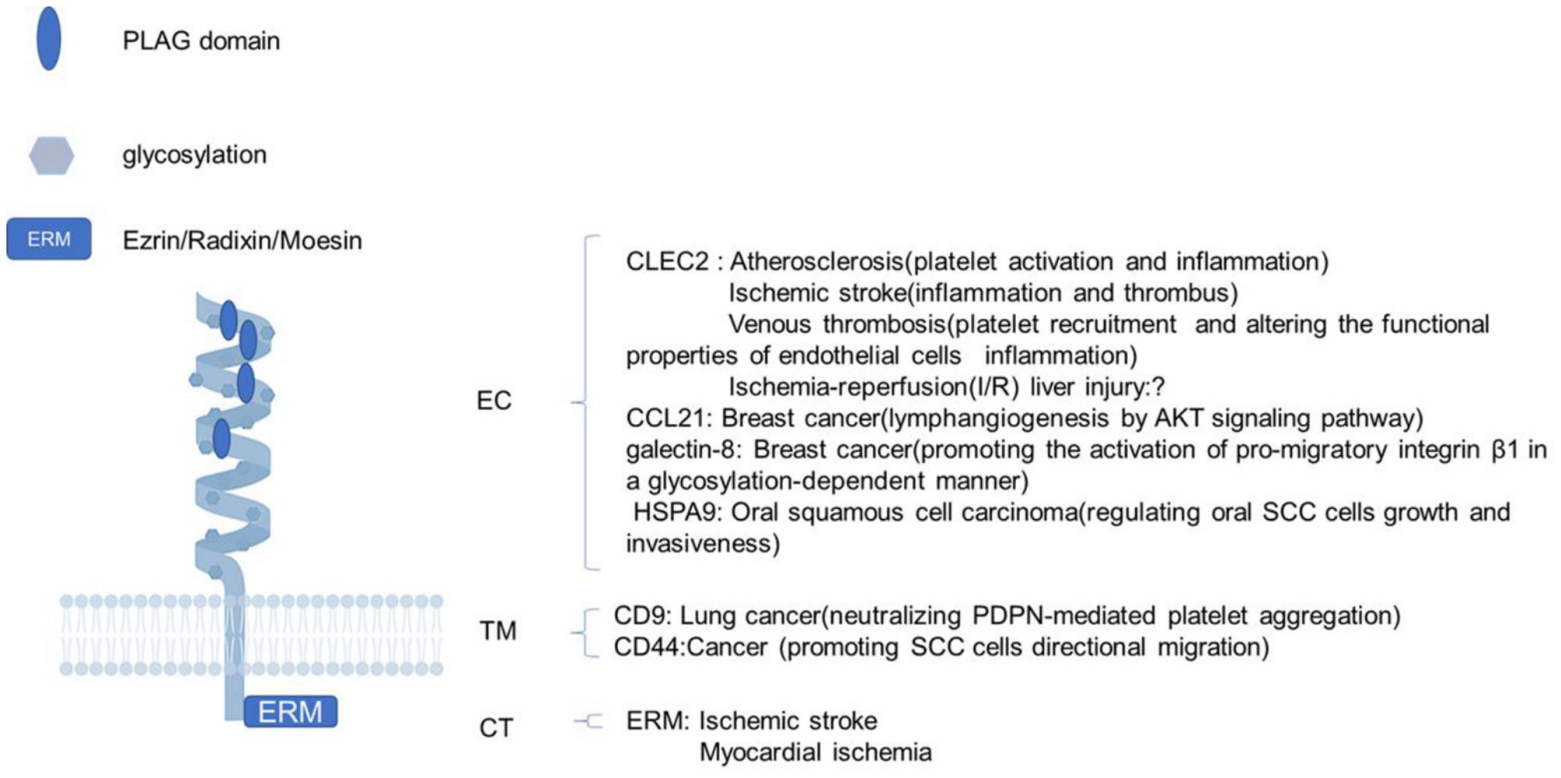
The structure of podoplanin and its functions with interacting proteins. Schematic representation of the molecular structure of podoplanin with a heavily glycosylated extracellular domain, a single transmembrane domain, and a short 9-amino acid cytoplasm. The ligands and biological processes during which the identified molecules interacting with podoplanin are presented. EC, ectodomain; TM, transmembrane region; CT, cytosolic domain; PLAG, platelet aggregation-stimulating.

Pdpn consists of a heavily O-glycosylated ectodomain, a hydrophobic membrane spanning domain, and a short cytoplasmic tail (CT) of only nine amino acids. Besides C-type lectin-like receptor 2 (CLEC-2), there are variable proteins interacting with Pdpn, such as CCL21, galectin-8, and heat-shock protein A9 (HSPA9) binding its ectodomain; CD9 and CD44 interacting with its transmenbrane domain; and ezrin, radixin, and moesin (ERM) binding to its CT. Through these interactions, Pdpn exerts various functions like platelet aggregation/activation, platelet biogenesis, immune surveillance, cytoskeleton rearrangement, and epithelial-mesenchymal transitions (EMTs) by protein–protein interactions for the lack of obvious enzymatic motifs (10–12) (Figure 1). Mostly, Pdpn is expressed on various cells, or at plasma membrane extensions, such as microvilli, filopodia, and ruffles, linking to the actin cytoskeleton to rearrange cytoskeleton and regulate cell motility. A fraction of Pdpn is localized in detergent-resistant membrane domains or raft platforms regulated by its CT and transmembrane domains, which appears to be necessary for Pdpn-mediated EMT and cell migration (13, 14). Besides, a soluble form of Pdpn (sPdpn) has recently been detected and investigated (15, 16). Cells ectopically or endogenously expressing Pdpn has been found to release extracellular vesicles (EVs) that contain Pdpn mRNA and protein. Pdpn incorporates into membrane shed microvesicles (MVs) and endosomal-derived exosomes (EXOs), and immunoelectron microscopy revealed its colocalization with the classical EV marker CD63 (15). Ovarian cancer cells express Pdpn themselves and also release Pdpn-rich EVs, both causing platelet aggregation, leading to venous thrombosis (16). Those Pdpn-EXO may contribute to sPdpn in circulating body fluid for Pdpn^+^ microparticles were detected in human body fluids including plasma and other liquids, which were quantitated using surface plasmon resonance, immunohistochemistry, and a double-antibody sandwich ELISA (17–20).

The Pdpn research was originally started from the cloning of highly metastatic NL-17 subclone from mouse colon 26 cancer cell lines and the establishment of 8F11 monoclonal antibody (mAb) that could neutralize NL-17-induced platelet aggregation and hematogenous metastasis. Pdpn was identified as the antigen of 8F11 mAb, whose ectopic expression brought cells the platelet-aggregating abilities and hematogenous metastasis phenotypes. From the 8F11 mAb recognition epitopes, Pdpn is found to contain tandemly repeated, highly conserved motifs, designated platelet aggregation-stimulating (PLAG) domains, which are associated with the CLEC-2 binding (21). Pdpn was discovered for the first time in rat and mice lungs, and on the surface of stromal cells in lymph nodes (LNs) in mice, which has been found to be expressed in a wide variety of cells later, such as lymphatic endothelial cells, tumor cells, osteocytes, choroid plexus epithelial cells, glial cells, and cancer-associated fibroblasts for its pleiotropic functions (7, 22).

## Pdpn signaling pathways

Among the many protein ligands of Pdpn, CLEC-2, and ERM proteins are studied comprehensively. CLEC-2 is a main receptor for Pdpn. The PLAG3 and PLAG4 domains of Pdpn are required for its binding to CLEC-2 (23, 24). The combination of CLEC-2 with the PLAG domains in the extracellular domain of Pdpn induces platelet activation and regulates inflammation through the Src, Syk, and SLP-76 kinase pathway (25, 26). Additionally, the interaction of Pdpn with CLEC-2 enhanced the interaction between Pdpn and ERM proteins and CD44, which activated Rho GTPase signaling pathway (27, 28). Both the interaction of Pdpn with CLEC-2 and with ERM are the two main pathways of cytoskeleton reorganization and inflammation regulation, which have been demonstrated to contribute to the occurrence and development of thrombotic diseases (29, 30). Studies show that Pdpn plays an important role in the functional regulation of immune cells. Following inflammatory or ischemic stimulation, Pdpn expression was upregulated in macrophages, microglia, and other immune cells, which influenced their motility and functionally phenotype transformation (31–33).

## Pdpn in atherosclerosis

Atherosclerosis is usually considered as a chronic inflammatory disease, which is the main root cause of thrombotic diseases characterized by lipid deposition in parts of the artery accompanied by smooth muscle cell (SMC) and fibrous matrix proliferation. Unstable atherosclerotic plaque rupture and following thrombus formation, or vascular stenosis lead to arteriosclerotic cardiovascular disease (ASCVD) resulting in high rate of mortality in the population (34). Platelet activation and aggression has a well-established role in the development and manifestation of atherosclerosis (35–37). Both CLEC-2 and Pdpn have been shown to bind to atherosclerotic lesions. CLEC-2 co-localized with vascular SMCs, while Pdpn was localized to SMCs and macrophages (38). Besides, Pdpn expression in SMCs and macrophages increased with atherosclerotic progression. However, in a rat model similar to the plaque erosion in human which contains relatively few inflammatory cells and more SMCs compared with plaque rupture, Pdpn was found to be overexpressed in endothelial cells, not in SMCs. Further exploration showed that vascular endothelial growth factor (VEGF)-A, which is expressed in SMCs, macrophages, and endothelial cells in the advanced atherosclerotic lesions, induced Pdpn expression. Therefore, it is speculated that VEGF-A from superficial SMCs stimulates endothelial Pdpn expression, which interacts with CLEC-2 to induce platelet aggregation and thrombus formation (39). The results remind us that at different stages of atherosclerosis, Pdpn expression varies in different cells and plays different roles. This partly might be explained by the fact that inflammatory stimulation upregulated Pdpn expression in macrophages, and Pdpn was expressed on inflammatory but not tissue-resident macrophages (31). Toll-like receptor (TLR) stimulation and some inflammatory cytokines activates Pdpn expression. Additionally, in advanced atherosclerotic plaque, Pdpn was detected in a membranous or cytoplasmic staining pattern, suggesting Pdpn may contribute to atherosclerosis development in both CLEC-2-dependent and independent manners (38). Pdpn is expressed in stromal myofibroblasts, which contribute to cell migration and invasion, suggesting a role of Pdpn in vascular remodeling and atherosclerotic progression in atherosclerotic plaques. On the other hand, inflammatory cytokines in plaque progression promote Pdpn expression in stromal cells and endothelial cells. Besides, adventitial lymphatics in the arterial walls protect against atherosclerosis, which are important in reverse cholesterol transport from atherosclerotic lesions (40). Pdpn was specifically associated with lymphatic endothelium number of adventitial lymphatics of human internal carotid artery, which demonstrated Pdpn may participate in atherosclerosis *via* regulating functions and regeneration of adventitial lymphatic vessels in atherosclerotic lesions (41). In a disturbed blood flow (d-flow) model, monocyte Pdpn was upregulated by d-flow, and the myeloid-specific Pdpn deletion mitigated the subendothelial accumulation of platelets and monocytes/macrophages, which ameliorated vascular inflammation (42) (Table 1).

**Table 1:** Pdpn in thrombotic diseases.

**Diseases**	**Species**	**Trend**	**Outcomes**	**Potential molecules**	**References**
Atherosclerosis	Human/Mouse	↑	Contributing to atherosclerosis development in both CLEC-2-dependent and independent manners.	CLEC-2, VEGF-A, inflammatory cytokines	Torres et al. (34), Kutkut et al. (40), Drozdz et al. (41)
Ischemic stroke	Human/Mouse	↑	High risk of stroke progression, poor prognosis, and death. Increased expression of CLEC-2 and Pdpn after I/R injury and protective effect of anti-Pdpn against I/R injury. Regulation of inflammatory cytokines through NLRP3? and thrombosis	CLEC-2, NLRP3?, RhoA/ROCK?	Zhang et al. (43), Wu et al. (44), Meng et al. (45), Zhao et al. (20), Qian et al. (46)
Venous thrombosis	Human/Mouse	↑	Anti-Pdpn antibody treatment and CLEC-2 deletion resulted in a reduction of thrombus formation. Pdpn overexpression was strongly associated with the amount of intratumoral thrombotic vessels and increased VTE risk in cancer patients. Anti-Pdpn antibody treatment inhibited platelet activation *in vitro* and decreased the incidence of VTE in mice.	CLEC-2	von Brühl et al. (47), Brill et al. (48, 49), Payne et al. (50), Kolenda et al. (51), Mir Seyed Nazari et (52, 53), Riedl et al. (54), Suzuki-Inoue (55), Wang et al. (56), Lee et al. (57), Sasano et al. (16), Sun et al. (58), Watanabe et al. (59), Tawil et al. (60), Zwicker (61)
Kidney ischemic injury	Human/Rats/Mouse	Glomeruli↓ renal interstitium↑	The increasing of urine Pdpn-to-creatinine ratio correlates with the onset of renal IRI. Significant decrease Pdpn expression in the renal glomerulus of diabetic kidney disease mice with an underlying chronic renal ischemia.	NF-κB?, mTOR?	Breiteneder-Geleff et al. (62), Weichhart et al. (63), Kezic et al. (64, 65), Zhang et al. (66), Chuang et al. (67), Kasinath et al. (68, 69), Yu et al. (70), Gao et al. (71)
Myocardial ischemia	Human/Mouse	↑	Upregulation of Pdpn in a heterogeneous cell population. Pdpn-neutralizing antibodies reduces inflammation post-MI without full suppression leading to heart function and scar composition improvement.	?	Mahtab et al. (72, 73), Douglas et al. (74), Cui (75), Loukas et al. (76), Noseda et al. (77), Popescu et al. (78), Aspelund et al. (79), Díaz-Flores et al. (80), Caporali et al. (81), Cimini et al. (82), Wakai et al. (83)
Ischemia-reperfusion liver injury	Mouse	↑	Activation of platelets.		Nakata et al. (84)

Much evidence confirmed the role of Pdpn in the development and manifestation of atherosclerosis mainly through inflammation and lymphatic vessel functional regulation pathways. CLEC-2 is the important partner for the role of Pdpn in atherosclerosis, however, other receptors and signaling pathways need to be explored.

## Ischemic stroke

Ischemic stroke is one of the most common thrombotic diseases, caused by a blood clot occluding one or multiple cerebral arteries, which means rapid recanalization of the occluded blood vessel is necessary for the treatment of acute ischemic stroke (AIS). However, even recanalization is successful, symptoms can still aggravate. This is called ischemia/reperfusion (I/R) injury, in which thrombotic and inflammatory pathways play a crucial role. Thus, ischemic stroke is recognized as a thromboinflammation disease (85). The Pdpn/CLEC-2 axis is thought to be a major regulator of thrombo-inflammatory disorders (86, 87). Therefore, we previously conducted a prospective observational study, including 352 AIS patients and 112 healthy controls. The results showed that plasma CLEC-2 (pCLEC-2) levels were associated with stroke progression and poor prognosis at 90 days. During 1 year follow-up, pCLEC-2 levels were also predictive for higher incidence of death and vascular events (43, 44). Further we examined the mechanism of Pdpn/CLEC-2 axis in cerebral ischemia injury using a mouse middle cerebral artery occlusion (MCAO) model. In this study, the expression of CLEC-2 and Pdpn increased after ischemia/reperfusion (I/R) injury and anti-Pdpn antibody pretreatment reduced infarct volume and attenuated the neurological deficits with a significant decrease of IL-18 and IL-1β, indicating a possible role of the Pdpn/CLEC-2 axis in the regulation of inflammation in ischemic stroke *via* modulating NLRP3 inflammasome (45). An upregulated Pdpn expression in reactive astrocytes in the ischemic model was observed, which might be a part of compensatory response to ischemic brain injury. This implied a remarkable role of Pdpn in astrocytes in ischemic brain injury, and cellular interactions among astrocytes, neurons, and microglia await to be elucidated further (20). Qian et al. reported the molecular mechanism of Pdpn neutralization inhibiting I/R-induced microglial activation using transcriptome sequencing analysis and found numerous inflammation-related signaling pathways were regulated by the anti-Pdpn treatment (46). Some upper proteins such as TRPM7 kinase might downregulate CLEC-2 to protect mice from acute ischemic disease without developing intracranial hemorrhage, which could provide us some clues on the mechanism of Pdpn/CLEC-2 axis in ischemic stroke (88). Both vascular and neurovascular interaction mechanisms may be involved, awaiting to be elucidated. Moreover, the interaction of the CT of Pdpn with the ERM protein family activates Rho GTPases. RhoA/ROCK signaling pathway in astrocytes is suggested to be crucial in neurogenesis and angiogenesis after cerebral ischemia, which indicates the crosstalk among podoplanin, ERM protein family, and astrocytes in ischemic stroke needs to be further studied (Table 1).

Pdpn contributes to the cerebral ischemia injury mainly through thrombosis and inflammation pathways. Its expression is upregulated after brain ischemia in various kinds of cells, some of which may not express Pdpn in normal conditions. However, the exact cellular interactions, vascular and neurovascular interaction mechanisms, and molecular signaling pathways remains to be elucidated.

## Venous thrombosis

Deep vein thrombosis (DVT) is a type of blood clot within deep veins, which is one of the most common venous thromboembolic disorders with a high mortality. Its underlying mechanisms still remain unclear, however, recent evidence has demonstrated that immune cells and inflammatory processes are involved in DVT initiation besides blood coagulation disorder (89). DVT is rich in red cells and fibrin, the formation of which involves the interaction of von Willebrand factor (vWF), platelets, neutrophils, and mast cells (47–49). In a murine DVT model of inferior vena cava (IVC) stenosis, it has been demonstrated that general inducible deletion of CLEC-2 or platelet-specific deficiency in CLEC-2 are protected against DVT. Also, anti-Pdpn antibody treatment resulted in a reduction of thrombus formation (50). The mechanisms have been suspected that the interaction of CLEC-2 in platelets and overexpressed Pdpn in the IVC wall induced venous thrombus formation. Highly distorted flow caused by IVC stenosis and following hypoxia led to upregulated Pdpn expression (51). However, Pdpn upregulation cannot only be a cause for thrombosis but might also be triggered by thrombus formation, which indicates both mechanisms may operate in parallel forming a positive feedback (Table 1). A recent study has demonstrated a role of CLEC-2 in cerebral venous thrombosis (CVT), an unusual manifestation of venous thrombosis. The results showed antibody (INU1-fab)-induced cooperative signaling of CLEC-2 and GPIIb/IIIa triggered a CVT-like thrombotic syndrome in mice. The authors speculated that INU1-fab alters the conformation of CLEC-2 and facilitates its interaction with an unknown ligand enriched in cerebral veins (90). Thus, Pdpn, a main ligand of CLEC-2 for platelet activation, was thought to be a candidate partner, as it is obviously upregulated in different inflammatory tissues including the brain, and can be shed from the cell surface to circulate in plasma (20, 91). However, it needs to be further explored.

A crucial role of the interaction between CLEC-2 and Pdpn in venous thrombosis has been revealed. Upregulated Pdpn expression was observed in DVT. The exact cellular expression and molecular signaling pathway remains to be uncovered, especially for CVT. Also, whether there are interactions between Pdpn and other receptors in venous thrombosis needs to be explored.

## Cancer-associated thrombosis

Moreover, Pdpn-associated platelet activation has been demonstrated to contribute to cancer-associated thrombosis, which are based on the upregulation of Pdpn on the cell surface of brain tumor cells. CATS trial reported that Pdpn overexpression was strongly associated with the amount of intratumoral thrombotic vessels and increased VTE risk in cancer patients. Platelet counts were lower and plasma D-dimer levels were higher in those with Pdpn-expressing brain tumors (52). Increased Pdpn expression in glioma cells coincides with the development of venous thrombo-embolism, which is correlated with laboratory evidence of coagulation activation by elevated D-dimer levels (54). CLEC-2-Pdpn interaction has been suggested to stimulate cancer-associated thrombosis in which thromboinflammation plays a crucial role. One hand, thromboinflammation induces ectopic podoplanin expression in vascular endothelial cells or macrophages; on the other hand, CLEC-2 depletion reduces levels of plasma inflammatory cytokines (55). Anti-Pdpn antibody treatment inhibited platelet activation *in vitro* and decreased the incidence of VTE in mice (56). In oral squamous cell carcinoma and ovarian cancer, the same results have been reported (16, 57). Hypermethylation of CpG islands in the Pdpn promoter was regulated by mutant isocitrate dehydrogenase (IDH) in glioma, which resulted in decreased Pdpn expression (58). Indeed, combination of IDH1 mutation and Pdpn expression in brain tumors can help identify patients at high risk of VTE (53, 59). Further exploration found Pdpn was released with exosome-like EVs shed from cells (60). Additionally, in a mouse model of systemic Salmonella Typhimurium infection, Pdpn was upregulated in monocyets and Kupffer cells (KCs) and its combination with CLEC-2 promoted the formation of infection-driven thrombosis in the liver (61, 92) (Table 1). Different forms of Pdpn participate in the formation of cancer-associated thrombosis, to which pdpn-mediated thrombosis, inflammation, and intratumoral vessel generation contributes. Besides CLEC-2, there may be other partners interacting with Pdpn in cancer-associated thrombosis.

## Kidney ischemic injury

Ischemia-reperfusion injury (IRI) is one of the most common causes of acute kidney injury (AKI), a serious and often deadly condition. Kidney IRI accounts for almost 50% of AKI cases, which is mediated by free radicals and reactive oxygen species (ROS) after periods of disrupted blood flow (68). Pdpn was named according to its expression in podocytes, mainly along their urinary surfaces, indicating a potentially functional role of Pdpn in kidney IRI (62). In a mouse model of kidney IRI, decreased Pdpn expression in the glomerulus and increased expression in the tubulointerstitial compartment of the kidney shortly after IRI was demonstrated. And the intensity of Pdpn in the tubulointerstitial compartment increased with the severity of ischemia, and the distribution of its expression changed over time (68). Moreover, an increase in the urine Pdpn-to-creatinine ratio was found to correlate with the onset of renal IRI. The researchers speculated that Pdpn was shed from the podocytes in an extracellular-vesicle form and expelled into the urine, which might be internalizated by the proximal tubule epithelium. Another hypothesis was spindle-shaped cells expressing Pdpn in the interstitium of the medulla might migrate to kidney from another organ, playing an important role in neovascularization during processes of kidney IRI. However, the exact mechanisms need to be further explored. Pdpn expression was significantly decreased in the renal glomerulus of diabetic kidney disease mice with an underlying chronic renal ischemia (70).

During the process, the activation of NF-κB signaling pathway in podocytes downregulated the expression of Pdpn, leading to increased podocyte apoptosis. Moreover, rapamycin, a kind of mTOR inhibitor, had a controversial role in the treatment of acute ischemic kidney injury. Some studies indicated a damage-promoting role of rapamycin during kidney IR injury (64, 65), while some reported a protective role of rapamycin against kidney IR injury (63, 65, 66). However, there was evidence on correlation between phosphorylated mTOR expression and Pdpn expression in esophageal squamous cell carcinoma and traumatic brain injury, which indicated Pdpn might participated in kidney IRI *via* mTOR pathway, awaiting to be explored (67, 71). Immune responses are involved in the pathophysiology of ischemic acute kidney injury (AKI) (93). In some immune diseases of kidney such as rescentic glomerulonephritis (GN), membrane Pdpn on fibroblastic reticular cells (FRCs) may play an important role in the pathogenesis. The effect of treatment with anti-Pdpn antibody was similar to that of FRC depletion by decreasing T-cell activation in the lymph node (LN), resulting in reduction of kidney injury (69). Fibroblastic reticular cells also maintain the integrity of high endothelial venules (HEVs) through interactions between Pdpn on the FRCs and CLEC-2 on platelets (9). Anti-Pdpn treatment led to disorganization of laminin fibers in the kidney LN, which was associated with remarkably reduced expansion of the lymphatic vasculature (69). Therefore, it is hypothesized that Pdpn on FRCs may contribute to ischemic kidney injury by immune regulation, which may be the future research contents (Table 1).

The role of Pdpn in kidney IRI remains unclear. Its mechanisms are complex. Both sPdpn and cellular form participate in the pathogenesis, in which NF-κB and mTOR signaling pathways have been implicated. Moreover, the role of Pdpn on FRCs in activation of T-cells and maintenance of the integrity of HEVs in kidney IRI needs to be explored.

## Myocardial ischemia (MI)

Myocardial ischemia (MI) is the commonest cardiovascular disease and one of the major causes of morbidity and mortality worldwide, in the pathogenesis of which inflammation and following heart tissue evolution play an important role. In the process, the growth and expansion of cardiac lymphatic vasculature in response to MI, is crucial for the transportation of extravasated proteins and lipids, inflammatory, and immune responses, as well as fluid balance (75, 76, 79). Therefore, Pdpn as a specific lymphatic marker, is thought to be vital in the cardiac development as well as the pathogenesis of MI. The function of Pdpn is crucial for epicardiac development and myocardial differentiation and its knockout shows a hypoplastic myocardium, atrioventricular valve abnormalities, and coronary artery abnormalities, which is partly correlated with reduced epithelial-mesenchymal transformation (EMT) caused by down-regulation of Pdpn (72, 74). Moreover, Pdpn deficiency results in hypoplastic sinus venosus myocardium including the sinoatrial node, which is also related to abnormal EMT due to up-regulated E-cadherin and down-regulated RhoA controlled by Pdpn (73). In the adult heart, Pdpn-positive cells only constitute <5% of the myocardial small cell population, which is only expressed by cardiac lymphatic endothelial cells in homeostatic conditions (94). However, after myocardial infarction (MI), Pdpn is upregulated in a heterogeneous cell population such as PDGFRα-, PDGFRβ-, and CD34-positive cells, besides lymphatic endothelial cells. Therefore, researchers thought Pdpn might be a sign of activation of a cohort of progenitor cells in different phases of post-ischemic myocardial wound repair. Inhibition of Pdpn by Pdpn-neutralizing antibodies reduces inflammation post-MI without full suppression leading to heart function and scar composition improvement. The increase of Pdpn-positive cells last from the acute (2 days) to the chronic phase of MI (2 weeks to 1 month) (82), which indicates a vital role of Pdpn in inflammation and wound repair after MI. Cimini et al. identified Pdpn as a potential cellular mediator of the lymphangiogenic and fibrogenic responses during different stages of myocardial wound repair after infarction (82). After injury, Pdpn is co-expressed by four kinds of cells such as PDGFRα-, PDGFRβ-, CD34-positive cells, and lymphatic endothelial cells which are responsible for regeneration, fibrosis, and inflammatory processes of the same pathologies. In the process, inflammation was thought to contribute to the recruitment of Pdpn-bearing LYVE-1-negative cells to the site of myocardial repair or the activation of Pdpn expression in responsive cell cohorts, which started the myocardial wound repair after infarction. At different stages of MI, Pdpn is expressed on various kinds of cells, for example, PDGFRα-positive cells during the whole process and PDGFRβ and CD34-positive cells at later stages of infarct healing in the mature scar. This means Pdpn plays multiple roles in the pathogenesis of MI. Cimini et al. reported the inhibition of the interaction between Pdpn and CLEC-2 expressing immune cells in the heart improved the cardiac performance, regeneration, and angiogenesis. In the model, Pdpn neutralizing antibody treatment induced recruitment of anti-inflammatory monocytes/macrophages and increased expression of anti-inflammatory cytokines (95).

Pericytes with PDGFR-β is very connected with Pdpn expression and transplantation of allogenic pericytes improves myocardial vascularization after MI resulting from the regulation of the endothelium in angiogenesis (81, 96). While mesenchymal stem cells (MSCs) expressing PDGFRα in the heart showed cardiomyocyte, endothelial, and smooth muscle lineage potential (77). *In vitro* differentiation of cardiac PDGFRα-positive cells brings out a lot of SMCs and endothelial cells only, indicating a predominant role of Pdpn in cardiac MSCs PDGFRα-positive cells in the vascular and mesenchymal compartments. CD34+telocytes expressed Pdpn after 15 days of MI, which supports cardiac growth, regeneration, renovation of connective tissue, and repair due to the unique communication with cardiac stem and progenitor cells (78, 80). Moreover, Pdpn expression significantly enhanced the migration of mesenchymal stromal cells (MSCs) and Pdpn-expressing MSCs extended processes into the endothelial cell layer, which could interact with circulating platelets (83) (Table 1).

In conclusion, cardiac ischemic injury induces upregulated and ectopic expression of Pdpn. The interaction of Pdpn and CLEC-2 or ERM proteins may participate in post-MI inflammatory response and cardiac repair through inflammation regulation, cytoskeleton reorganization, and lymphangiogenic and fibrogenic responses. The exact mechanisms remain unclear. And the interaction of Pdpn with other partners in cardiac ischemic injury needs to be further explored.

## Ischemia-reperfusion (I/R) liver injury

Hepatic I/R injury is usually associated with surgical procedures, trauma, liver transplantation, or resection as a consequence of interrupted blood supply to the liver, which leads to liver dysfunction and failure, as well as multiple organ failure (97, 98). Kupffer cells (KCs) and platelets were reported as two main roles in the procedure (99–101). Nakata et al. revealed Pdpn expression in the cytosol of hepatocytes in the post-ischemic liver and KC depletion weakened the Pdpn expression, which suggested that activated KCs regulate the expression of Pdpn in hepatocytes after I/R without clear mechanisms (84). Moreover, the authors demonstrated in the acute phase of hepatic I/R injury, the binding of CLEC-2 on the cell surface of platelets to Pdpn in hepatocytes activated platelets in the hepatic sinusoid (84). Therefore, the crosstalk among podoplanin, KCs, and platelets in hepatic I/R injury needs to be further studied (Table 1).

## Conclusions and perspectives

Pdpn, as an important glyprotein, has multiple interacting proteins in various tissues and organs, demonstrating its pleiotropic functions, especially a role in thrombosis and inflammation. Thrombosis and inflammation contribute to the pathogenesis of thrombotic diseases, such as atherosclerosis, ischemic stroke, venous thrombosis, acute kidney and liver ischemic injury, and myocardial ischemia. Evidence showed that after ischemia, Pdpn can be acquired over time by a heterogeneous cell population such as SMCs, endothelial cells, astrocytes, pericytes, MSCs, telocytes, and so on, which may not express Pdpn in normal conditions. However, the exact mechanisms of Pdpn in such ischemic diseases have not clearly been demonstrated. Pdpn in different cells plays different roles such as thrombosis, inflammation, vascularization, lymphagiogenesis, growth, and regeneration. However, many issues remain to be elucidated further; for instance, cell/stage-specific effects of Pdpn and according molecular mechanisms, and the relevance of anti-Pdpn treatment on ischemic diseases, especially ischemic stroke, venous thrombosis, and myocardial ischemia. The solutions to these issues can provide a new target of treating thrombotic diseases from bench to clinical translation.

## Author contributions

YH: writing-original draft, visualization, and data curation. ML and YW: data curation, visualization, and resources. CZ: validation, investigation, and resources. YC: writing—review, editing, and funding acquisition. XZ: conceptualization, methodology, project administration, writing—review, editing, funding acquisition, and supervision. All authors have carefully read and confirmed the final manuscript. All authors contributed to the article and approved the submitted version.
